# Impact of Barley Malt with Different Row-Types on the Volatile Compounds in Beer

**DOI:** 10.3390/foods14122010

**Published:** 2025-06-06

**Authors:** Jinglong Zhang, Ziqiang Chen, Yongxin Zhang, Zhenbao Shi, Jian Lu, Dianhui Wu

**Affiliations:** 1 Key Laboratory of Industrial Biotechnology, Ministry of Education, Jiangnan University, Wuxi 214122, China; 15085385906@139.com (J.Z.); 6220209065@stu.jiangnan.edu.cn (Z.C.); 6220209057@stu.jiangnan.edu.cn (Y.Z.); 6230210024@stu.jiangnan.edu.cn (Z.S.); jlu@jiangnan.edu.cn (J.L.); 2 National Engineering Research Center of Cereal Fermentation and Food Biomanufacturing, Jiangnan University, Wuxi 214122, China; 3 Jiangsu Provincial Research Center for Bioactive Product Processing Technology, Jiangnan University, Wuxi 214122, China; 4 School of Biotechnology, Jiangnan University, Wuxi 214122, China

**Keywords:** two-row malt, six-row malt, volatile compounds, beer flavor, correlation analysis, flavor precursors

## Abstract

As the primary raw material for beer production, barley is classified into two-row barley and six-row barley. The nutrient content is different in the different row-types of malts, and the beer volatile compounds (VCs) will be influenced when using them. The results showed that the wort produced from six-row malt contained more fermentable sugars (FSs) (26.3%) compared to two-row malt, and their free amino acid (FAA) profiles were apparently different. These differences were translated into variations in the VCs of beer. Six-row malt beer contained a higher content of total VCs (6354.80 μg/L), and most of the content of individual VC (66.7%) was significantly higher than two-row malt beer. In contrast, two-row malt beer showed a higher content of 1-propanol, ethyl caprate, and octanoic acid isoamyl. Eight key volatiles contributed to the differences in beer flavor, and these differences (62.5%) were related to the major amino acids (alanine, arginine, phenylalanine, tyrosine, and threonine). This study clarified how barley with different row-types affected beer VCs and offered guidance for selecting raw materials in beer production.

## 1. Introduction

Beer is a globally popular alcoholic beverage [1]. Barley malt serves as the primary raw material for beer brewing, constituting approximately 90% of total malt production [2]. Based on the types of barley ridges, barley can be classified into two-row barley (*Hordeum distichon* L.) and six-row barley (*Hordeum vulgare* L.). Studies have shown that six-row malt exhibits a higher protein/amylose ratio and more enzymes compared to two-row malt [3,4]. Beer brewing typically requires malt with lower protein and higher starch content, making two-row varieties preferable. The nutritional composition of different malt types directly influences aromatic profiles and odor-active compounds in beer. It has been reported that malt type affects the chemical composition, volatile compounds (VCs), and sensory profile of beer [5,6]. However, it remains unclear how pure two-row or six-row malt and their mixtures affect: (a) wort extracts such as fermentable sugars (FSs) and free amino acids (FAAs); (b) VCs in the finished beer.

VCs are direct determinants of the aroma quality of beer and the key factors in determining the sensory quality of beer. They are mainly influenced by the way that beer is produced and the raw materials used for production, such as yeast, malt, and hops [7,8]. The type and quality of malt have a significant impact on the yeast metabolism and VCs in beer [5]. A study of six different sources of malt showed that the metabolites in malt from different sources had about 23% variation and that the metabolites in the beers produced from these malts displayed approximately 61% variation [9]. In addition, the sensory descriptions of beer brewed from different varieties of malt are quite different, since the barley genotype has a significant effect on beer flavor, and some sensory descriptors are environment-specific [10]. This suggests that the differences in metabolites of barley malt are not only retained but may also be amplified during the brewing process, resulting in significant variations in the final beer.

Instrumental analytical techniques such as gas chromatography–mass spectrometry (GC-MS) and gas chromatography electronic nose (GC-E-nose) are widely used for flavor analysis of foods [11]. GC-MS can characterize and quantify VCs and has the advantages of strong separation and identification ability [12,13]. However, GC-MS cannot provide sensory evaluation of beer, whereas electronic sensing technologies such as electronic nose (E-nose), which simulate human sensory perception, have recently been widely applied in the field of food science. The E-nose mimics the human olfactory organ to recognize various odors and expresses small changes in VCs as sensor differences [14]. GC-E-nose is developed based on the separation principle of gas chromatography and can reveal more information about VCs than sensor-type E-nose [15]. One of the major research trends in the field of food flavor is the application of E-nose in combination with GC-MS to study the aroma characteristics of substances [11,16]. In summary, GC-MS enables accurate qualitative analysis of VCs in food, while GC-E-nose allows for rapid and precise identification of unknown odor types.

In this study, GC-E-nose and GC-MS combined with multivariate statistical analysis were applied to explore the effects of barley malt with different row-types on VCs in beer. The key VCs that caused the differences in beer were investigated through the analysis of nutrients in barley malt. The results of this study will provide a theoretical basis for a deeper understanding of the effects of barley malt on beer volatiles and have a certain reference value for the technological development and quality control of beer.

## 2. Materials and Methods

### 2.1. Samples and Reagents

European malt is a preferred choice among brewers. France, being the leading producer of beer barley in Europe, is also the top exporter of malt globally; additionally, France uses six-row barley to make malt [17]. Consequently, this study selects French barley as the research material. Two-row malt (French malt, Planet) and six-row malt (French malt, FARO) were provided by Nongken Malt Co., Ltd. (Sheyang, Jiangsu, China). Magnum hops were purchased from Libo Zhaohe Hops Co., Ltd. (Beijing, China); Cascade hops were purchased from Yakima United Hops Co., Ltd. (Yantai, Shangdong, China). *Saccharomyces cerevisiae* ALE-514 was purchased from Mauribrew Co., Ltd. (Rolleston, New Zealand). The hops and yeast were stored in a refrigerator at 4 °C.

Glucose standard, fructose standard, maltose standard, sucrose standard, maltotriose standard, and DNS reagent were purchased from Beijing solarbio science & technology Co., Ltd. (Beijing, China). Na_2_HPO_4_, KH_2_PO_4_, ninhydrin hydrate, KIO_3_, ethanol, and glycine were purchased from Shanghai Meryer Biochemical Technology Co., Ltd. (Shanghai, China). In addition, 2-Octanol and C_6_-C_30_ n-alkane standards were purchased from Shanghai Aladdin Biochemical Technology Co., Ltd. (Shanghai, China). Trichloroacetic acid was purchased from Sinopharm Chemical Reagent Co., Ltd. (Shanghai, China).

### 2.2. Wort Preparation

The experiment samples were divided into three groups (100% two-row malt, equally mixed two-row malt, and six-row malt, 100% six-row malt). The saccharification method was modified based on the method by Zhang et al. [18]. The mashing process was conducted in a temperature-controlled mash bath (Model: LB-12, Lochner Labor). When the water in the saccharification cup was raised to 48 °C, the crushed malt was added at a ratio of 1:3.5 for 10 min; the temperature was then raised to 63 °C at a rate of 1 °C/min for 60 min. Subsequently, it was raised to 72 °C at a rate of 1 °C/min for 10 min and then raised to 78 °C for 10 min. The wort was filtered and sparged with 80 °C water to make the wort concentration 11 °P. The obtained wort was boiled for 60 min, and 0.4‰ Magnum hops and 0.4‰ Cascade hops were added when it was just boiled and after 50 min, respectively. The initial extract of the wort was adjusted to 12 °P.

### 2.3. Beer Fermentation

A total of 300 mL wort was poured into a 500 mL sterile fermentation bottle and activated yeast was added. The number of yeast was controlled at about 1 × 10^7^ CFU/mL. The main fermentation temperature was maintained at 20 ± 1 °C. The weight loss of the fermentation system was monitored every 12 h, and the flask was shaken every 8 h for 30 s until the daily weight loss of beer was reduced to less than 0.2 g, and the main fermentation was completed. Then, it was stored in a refrigerator at 4 °C for 7 days, and the fermentation was completed.

### 2.4. Determination of Physicochemical Parameters

The color and α-amino nitrogen (α-N) content of wort were determined according to the Chinese national standard QB/T 1686-2008 [19]. The reducing sugar was determined by the DNS method, and the pH value was determined by a digital pH meter (Mettler Toledo, Shanghai, China).

The alcohol content, real extract, and real fermentation degree (RDF) of beer were determined according to the Chinese national standard GB/T 4928-2008 [20]. The pH value was measured by a digital pH meter (Mettler Toledo, Shanghai, China).

### 2.5. Wort of FSs and FAAs Analysis

According to the method of Qi et al. [21], fructose, glucose, maltose, sucrose, and maltotriose in wort were determined by high-performance liquid chromatograph (Waters 1525EF, Waters, Milford, CT, USA). Sample pretreatment: 10 mL of wort was centrifuged at 7000 rpm/min for 10 min, and the supernatant was passed through a 0.22 μm filter membrane. The detection conditions were as follows: C_18_ column (4.6 mm × 250 mm, 4 μm, Waters), mobile phase 0.1 mol·L^−1^ ammonium acetate: acetonitrile (83: 17, v:v), column temperature 35 °C, ultraviolet detector (254 nm), injection volume of 10 μL, flow rate of 1 mL·min^−1^. FSs were quantified according to the standard curve for each sugar.

FAAs in wort were analyzed by the automatic amino acid analyzer S433D (Sykam, Eresing, Germany). Sample pretreatment: the samples were diluted 2-fold with 10% (v/v) trichloroacetic acid and equilibrated at room temperature for 1 h. The samples were centrifuged at high speed (10,000 rpm/min, 10 min), and the supernatant was filtered through a 0.22 μm microporous membrane. The detection conditions were as follows: Agilent Hypersil ODS column (250 mm × 4.6 mm, 5 μm, Agilent, Santa Clara, CA, USA). The injection volume was 10 μL, the flow rate was 1.0 mL·min^−1^, and the temperature of the column was 40 °C. The ultraviolet detector was operated in dual-wavelength mode, with the main detection wavelength of 338 nm and the characteristic detection wavelength of proline at 262 nm, and the mobile phases consisted of two buffer solutions: A was a mixture of sodium acetate (27.6 mmol/L), tetrahydrofuran, and triethylamine (v/v/v, 500:2.5:0.11, pH = 7.2); B was a mixture of sodium acetate (80.9 mmol/L), acetonitrile, and methanol (volume ratio: 1:2:2, pH = 7.2). The gradient elution procedure was carried out in the following chronological order: the initial phase (0 min) A:B = 92:8; adjusted to 50:50 at 17 min; switched to 0:100 at 20.1 min; and the initial ratio of 92:8 was restored at 24.0 min. Compound identification was achieved by comparison with retention times of the standards, and quantitative analyses were performed by the external standard method.

### 2.6. GC-MS Analysis

The VCs in the samples were extracted by headspace solid phase microextraction (HS-SPME), and the VCs in the samples were identified by GC-MS (Trace GC-1310-ISQ LT; Seymour Fisher Technologies Inc., Waltham, MA, USA). The samples were incubated at 55 °C for 15 min and then extracted for 30 min. After extraction, the extraction head was quickly inserted into the GC inlet and desorbed at 250 °C for 1 min. The capillary column was TG-WAXA column (60 m × 0.25 mm × 0.25 μm). The chromatogram was recorded by monitoring the total ion current in the range of m/z 20~450. The chromatographic temperature program was set as follows: the initial temperature was 40 °C, maintained for 1 min, then increased to 180 °C at a rate of 3 °C/min, and finally increased to 230 °C at a rate of 20 °C/min, maintained for 15 min. The detector temperature was 250 °C. The carrier gas was helium (99.999%), and the flow rate was 1.2 mL/min. The ion source temperature of the mass selective detector was 260 °C. The electron ionization energy was 70 eV and the transmission line temperature was 230 °C. The chromatograms were recorded in the mass range of 29–450 μm. The compounds were characterized by the National Institute of Standards and Technology mass spectrometry library standard spectrum and retention index (RI) reported in the literature. The relative quantitative analysis of VCs in beer was carried out with 2-octanol as the internal standard.

The ratio of the relative odor activity value (rOAV) to the maximum rOAV (ROAV) of each VC can evaluate the contribution of a single volatile compound to the overall aroma by the relative concentration of a single volatile compound [22]. The calculation formula of rOAV is rOAV = C_i_/OT_i_. In the formula, OT_i_ is the threshold value (i) of the compound in water or alcohol; C_i_ is the relative content of VCs. The ROAV of volatile compound (i) was calculated as ROAV_i_ = (rOAV_i_/rOAVmax) × 100 [22].

### 2.7. GC-E-Nose Analysis

The method of GC-E-nose was referred to by Ma et al. [23]; based on gas chromatography fast electronic nose analysis, Herakles II (Alpha MOS, Toulouse, France) was equipped with two parallel mode chromatographic columns with different polarities: DB-5 (10 m × 0.18 mm × 0.4 m) and DB-1701 (10 m × 0.18 mm × 0.4 m), and two flame ionization detectors (FIDs). The headspace extraction was performed by adding 4 mL of sample into a 20 mL headspace vial at a constant temperature of 45 °C for 20 min. Injection parameters were 200 °C, 125 μL/s, and injection time 25 s. The acquisition was carried out in separation mode (10 mL/min) at 40 °C for 30 s. Separation of analytes was achieved by a temperature program: starting at 40 °C, then increasing at a rate of 1 °C/s to 120 °C, followed by a rate of 3 °C/s to 250 °C, and held for 60 s. The carrier gas was hydrogen. The compounds were introduced in a static headspace mode and pre-concentrated on a Tenax trap. The HS sampler (XYZ, Alpha MOS, Toulouse, France) was used for automatic sampling. The software used for data collection was AlphaSoft (https://www.alphasoftware.com/download-page) (Alpha Soft, Inc., Burlington, MA, USA). Then, the principal component analysis (PCA) chart and radar chart were generated by GC-E-nose software (Vocal 1.1) based on differential VCs with peak area differences > 0.85 in the GC-E-nose.

### 2.8. Statistical Analysis

The results were expressed as mean ± standard deviation, and all results were in triplicate. All data were processed using SPSS 26.0 (IBM Corp., Armonk, NY, USA). One-way analysis of variance (ANOVA) was used to compare the mean values of each group by Duncan’s test, and *p* < 0.05 was considered statistically significant. The correlation heatmap between groups and point stick heatmap was generated by R 4.4.1. The partial least squares discriminant analysis (PLS-DA) model was generated by SIMCA 14.1. The remaining images were generated by Origin Pro 2022 (OriginLab Corporation, Northampton, MA, USA).

## 3. Results and Discussion

### 3.1. Analysis of FSs and Amino FAAs in Barley Malt

#### 3.1.1. Physicochemical Analysis

All wort concentrations were adjusted to 12 °P before fermentation to ensure that the wort had the same soluble solid content. As shown in Table S1, the content of α-N in six-row malt wort (SW) (244.63 mg/L) was significantly higher than that in two-row malt wort (TW) (229.38 mg/L), and the equal mixture of the two malts’ wort (MW) (233.97 mg/L) (*p* < 0.05). Moreover, with the increase in the content of six-row malt, there was a significant increase in the content of reducing sugars in the wort, which increased by 4.90%. This could be attributed to the fact that six-row malt is richer in enzymes and proteins than two-row malt [3]. The pH in the wort was in the range of 5.56–5.58, but the color in TW (7.47 EBC) was significantly higher than that in MW (6.87 EBC) and SW (6.77 EBC). As far as the malt is concerned, the flavor of the beer is mainly influenced by the sugar, protein, and FAA content of the malt [9,24].

#### 3.1.2. Analysis of FSs in Wort

**Figure 1:**
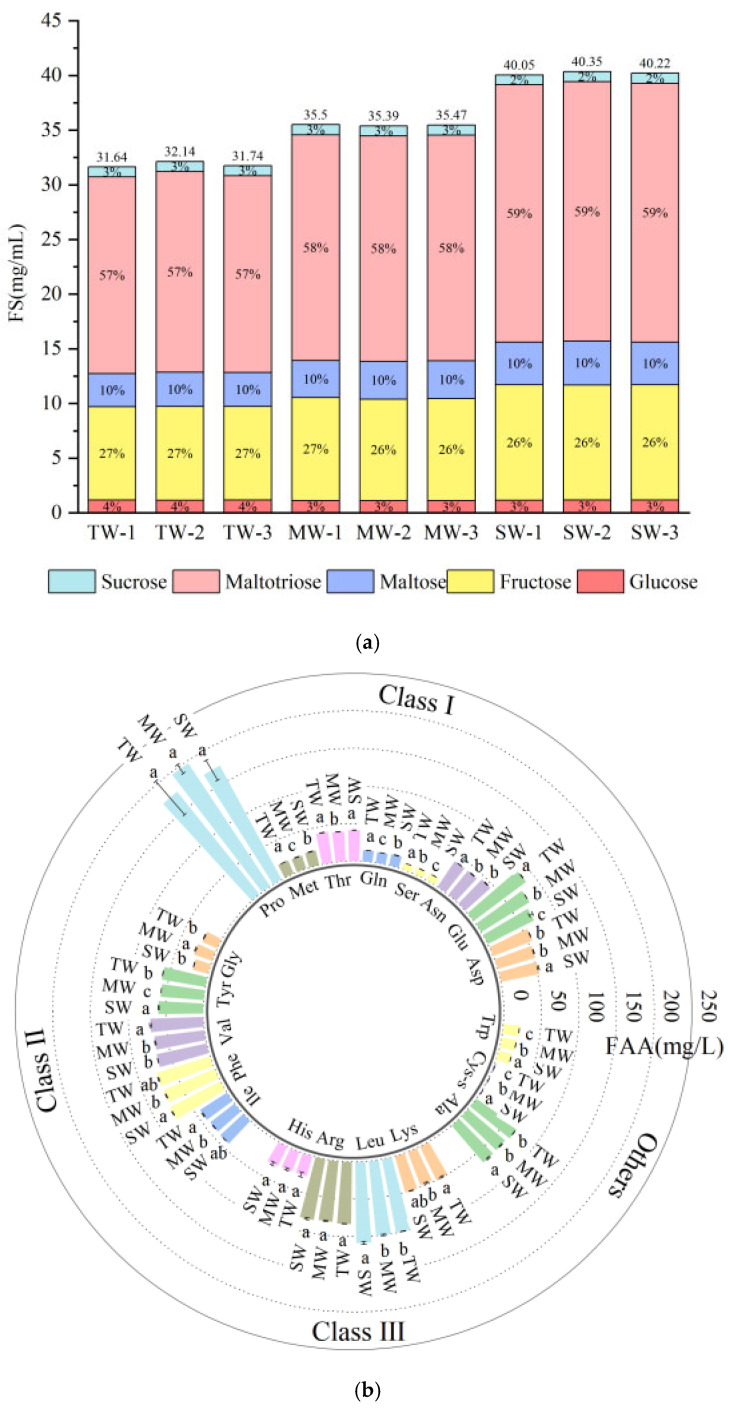
Stacked bar chart of FSs in wort (**a**) and circumplex of FAAs in wort (**b**). FS: fermentable sugar; FAA: free amino acid; TW: two-row malt wort; MW: equally mixed two-row malt and six-row malt wort; SW: six-row malt wort. a–c indicated significant differences between values at *p* < 0.05.

Sugars in wort accounted for over 90% of the extract concentration, with the majority (75–78%) being FSs [25]. As shown in Table S2, SW had the highest fermentable sugar content, followed by MW and TW with 39.29 g/L, 34.53 g/L, and 30.93 g/L, respectively, which could be attributed to the fact that six-row malt was richer in amylase, which promotes the hydrolysis of starch in the wort. The highest concentration of maltotriose was found in the wort, followed by glucose, maltose, fructose, and sucrose, which were in similar proportions (Figure 1a), indicating that the type of malt rows mainly affects the content of volatiles in the beer and does not influence its composition. Maltotriose (58.6–60.2%) was dominant among the FSs examined [24], and its contents in SW and MW were 23.64 g/L and 20.63 g/L, respectively, which were 30.4% and 13.8% higher than TW.

#### 3.1.3. FAAs Analysis in Wort

The compositional structure of FAAs in wort has an important influence on the flavor of beer [26]. As far as amino acids are concerned, the nitrogen metabolism of yeast during fermentation depends on the total concentration of FAAs and the concentration of individual amino acids [27]. FAAs in wort were determined, and the concentrations of 20 kinds of FAAs were listed in a classification (Table S3). The classification of FAAs was based on the nature of the amino acids [28].

The total FAA content and individual FAA content in the wort were similar to those reported in the literature [29]. SW had the highest total FAA content, followed by MW and TW with 1059.7 mg/L, 1053.9 mg/L, and 1047.5 mg/L, respectively. The composition and concentration of FAAs in the wort were shown in Figure 1b. It is apparent that the addition of six-row malt did not alter the relative proportion of each FAA in the wort. This can be attributed to the fact that the two-row and six-row malts were both barley malts, which supply similar amounts of nitrogen during the protein resting phase. Although class I amino acids exhibited high levels in FAAs, their concentration was relatively unimportant to the quality of the beer because yeast can synthesize or directly take up their ketoacid analogs from the wort to produce by-products of their synthesis through gluconeogenesis and transamination [26,28]. The concentration of class II and III amino acids, on the other hand, can have a dramatic effect on the flavor of the beer. When exogenous class II amino acids are depleted, yeast synthesis of their keto acid analogs from sugar is accompanied by the production of carbonyl by-products such as diacetyl, resulting in off-flavors in beer [26]. The utilization of class III amino acids by yeast depends on adequate exogenous supply, so the deficiency of class III amino acids will seriously affect the nitrogen metabolism of yeast and, consequently, the flavor quality of beer [26]. The results showed that the concentrations of glycine, tyrosine, valine, phenylalanine, isoleucine, histidine, arginine, leucine, and lysine in the wort exhibited significant variations. Valine (70.8 mg/L), isoleucine (42.6 mg/L), and leucine (108.0 mg/L) were present in the highest amounts in TB. In contrast, SW had the highest concentrations of tyrosine (59.7 mg/L) and phenylalanine (75.5 mg/L). The branched-chain amino acids valine and leucine are conducive to the formation of isobutanol and isopentyl alcohol, respectively, and subsequently promote the formation of the corresponding ethyl esters [30]. The MW sample had the highest concentration of glycine (25.7 mg/L). Previous studies have indicated that glycine can prolong the yeast’s ethanol production phase [31], which may potentially affect the rate of metabolism in yeast. Additionally, SW also had the highest concentrations of cysteine (1.8 mg/L), tryptophan (21.7 mg/L), and alanine (67.3 mg/L). Among these, phenylalanine can be converted to phenylacetaldehyde, while tryptophol has been associated with unpleasant aromas [32]. Apparently, these worts differ in the composition of FAAs, which were key precursors in the yeast synthesis of higher alcohols and aldehydes (e.g., isoamyl alcohol, acetaldehyde), and therefore, there should be differences in the flavor of the beers fermented from them.

### 3.2. Flavor Analysis of Beer

#### 3.2.1. Physicochemical Parameters of the Beer

As shown in Table S4, there was no significant difference in alcohol content (4.18%vol–4.22%vol), real extract (4.97–5.18%), RDF (57.26–58.29%), and pH (4.61–4.71) of the beers fermented from the three wort types.

#### 3.2.2. Analysis of VCs by GC-E-Nose

**Figure 2:**
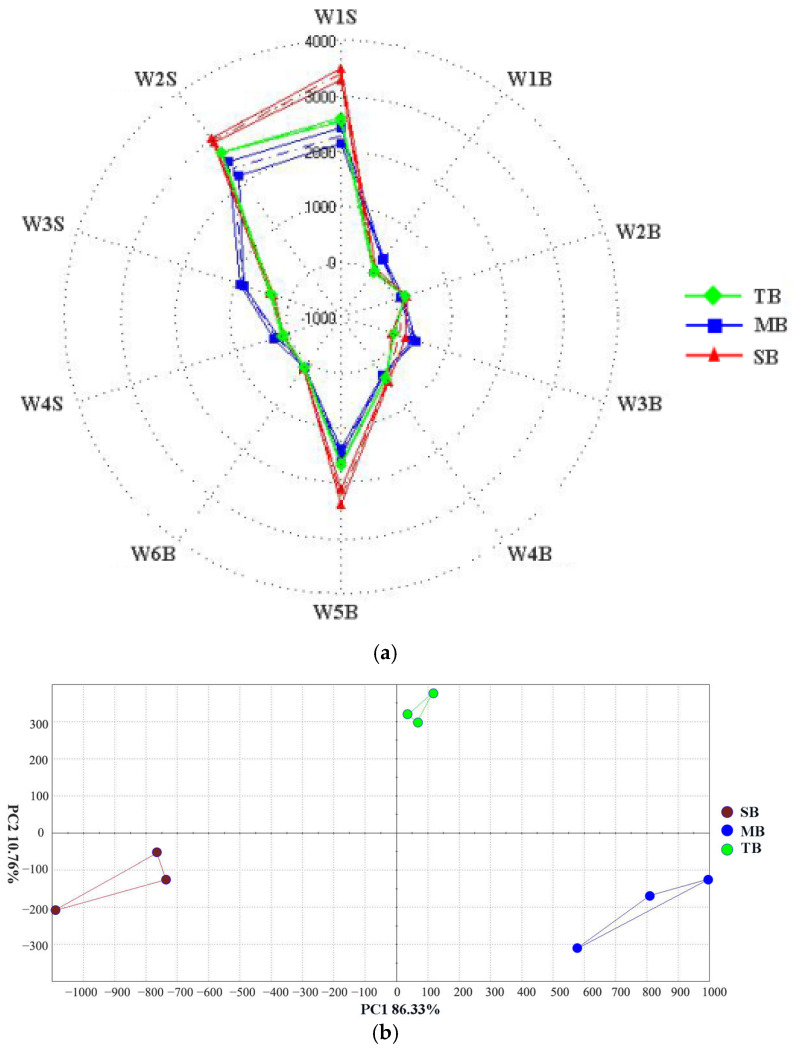
Radar map (**a**) and PCA results (**b**) based on the dates of GC-E-nose in different beer samples. TB: two-row malt beer; MB: mixed malt beer; SB: six-row malt beer.

The GC-E-nose integrates the functions of GC and E-nose, which enables an objective and fast analysis of the aroma substances of different samples by simulating human senses [33]. Two-row malt beer (TB), mixed malt beer (MB), and six-row malt beer (SB) beers were analyzed by GC-E-nose. PCA plots and radar plots were constructed from the GC-E-nose data, as shown in Figure 2.

The radar map (Figure 2a) demonstrated that the radar map profiles of TB, MB, and SB were different. The concentration of differential volatiles was higher in SB, which may be because more carbon and nitrogen sources in the wort of six-row malt promoted the production of VCs in the beer. In addition, the radar map profile of MB differed from both TB and SB, suggesting that MB has a unique flavor profile. PCA is an unsupervised technique that identifies differences between datasets, which can quickly outline the differences between samples [34]. The cumulative variance contribution of PCA (Figure 2b) was 97.09% (PC1: 86.33%, PC2: 10.76%), which indicated that the PCA results could effectively explain the flavor differences between samples. The distances between the samples in the PCA plots reflect the magnitude of their differences [35], and the samples were obviously located in different positions in the PCA plot, with TB, MB, and SB located in the first, third, and fourth quadrants, respectively, indicating that there were obvious differences in the flavor characteristics among the three samples.

#### 3.2.3. Analysis of VCs by GC-MS

Based on the GC-MS analysis of VCs in TB, MB, and SB, as presented in Table 1, a total of 60 VCs were identified, comprising 21 alcohols, 20 esters, 5 acids, 5 aldehydes, 5 ketones, and 4 other compounds. The total VC content was found to be the highest in SB at 6354.80 μg/L, followed by TB and MB with concentrations of 5759.76 μg/L and 5719.47 μg/L, respectively.

**Figure 3:**
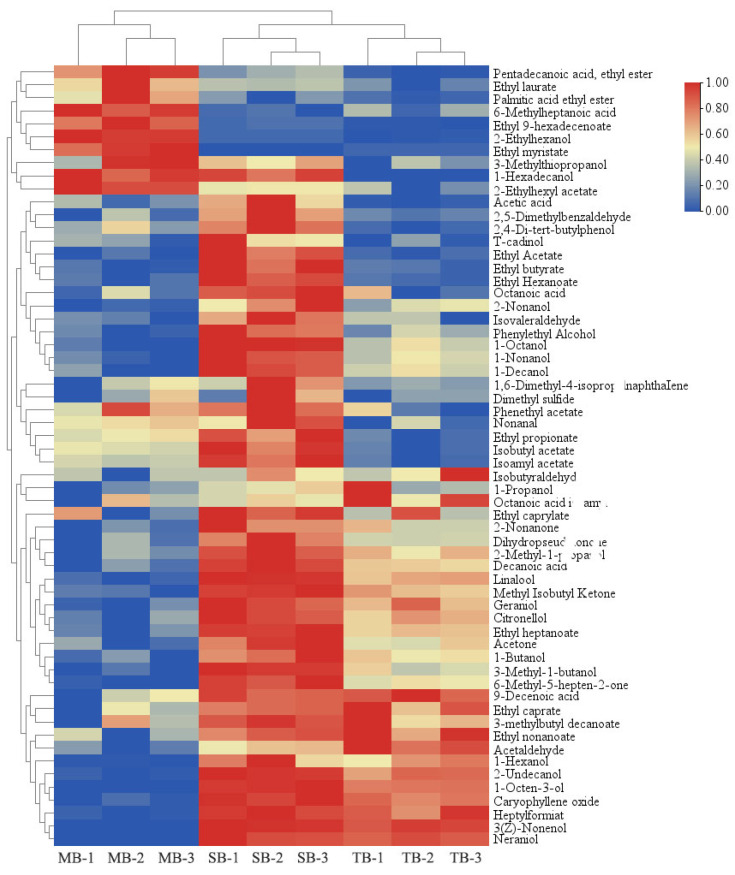
Flavor heatmap of different beer samples based on GC-MS. TB: two-row malt beer; MB: mixed malt beer; SB: six-row malt beer.

To visualize the flavor differences among the three beers, the data of the 60 identified VCs were normalized, and the results were presented using a flavor heatmap (Figure 3). It can be observed that the concentrations of most compounds in SB were significantly higher than those in TB and MB, which may be because the main flavor precursors (FAAs and FSs) in the latter two beers were similar in composition and structure, but the concentrations of flavor precursors in SB were higher. Apparently, the heatmap results were consistent with the results of the GC-E-nose analysis.

The VCs in the beer were mainly alcohols (43.2–45.2%), esters (33.5–36.1%), and acids (18.7–19.3%). The VCs (66.7%) in SB were significantly higher than that in TB (*p* < 0.05), which suggested that the six-row malt facilitates the formation of flavor compounds in the beer in comparison with the two-row malt. Beers fermented with a mixture of malts, on the other hand, showed a decrease in most compounds and a significant increase in the content of eight alcohols and esters, including 2-ethylhexanol, 1-hexadecanol, palmitic acid ethyl ester, 2-ethylhexyl acetate, pentadecanoic acid ethyl ester, ethyl laurate, ethyl 9-hexadecenoate, and ethyl myristate. This was what contributed to the flavor distinction of MB from TB and SB.

The higher alcohols are the most representative compounds in beer and are mainly produced by amino acids via the Ehrlich pathway and the sugar metabolism pathway [28,36]. Among the alcohols identified, 3-methyl-1-butanol (37.9–40.0%), phenylethyl alcohol (47.2–48.3%), and 2-methyl-1-propanol (3.9–4.1%) were the major alcohol compounds, which were considered the main higher alcohols in the beer [37]: 2-Methyl-1-propanol, 3-methyl-1-butanol, and phenylethyl alcohol have a sweet aroma, wine aroma, and brandy-like aroma, respectively, which are mainly higher alcohols converted from valine, leucine, and phenylalanine through the Ehrlich pathway [38]; 3-Methyl-1-butanol (1146.50 μg/L), phenylethyl alcohol (1353.47 μg/L), and 2-methyl-1-propanol (115.15 μg/L) had the highest content in SB, which may relate to the relatively high content of reducing sugars and corresponding amino acids in its wort; 2-Ethylhexanol (77.63 μg/L), 3-methylthiopropanol (3.03 μg/L), and 1-hexadecanol (12.37 μg/L) were the highest in MB. Among them, 3-methylthiopropanol is an important sulfur-containing higher alcohol that gives beer its characteristic flavor [39].

Esters are mainly produced by enzymatic metabolic reactions of higher alcohols under the action of acyl coenzyme A [18], which can give the beer a pleasant aroma, such as a fruity and floral aroma. The main esters identified were ethyl caprylate (24–27%), ethyl caprate (18–24%), ethyl laurate (15–17%), and isoamyl acetate (5–7%). Of these, isoamyl acetate and ethyl caprylate were associated with banana and fruit flavors, respectively, and were common esters found in beer [16]. Their contents in six-row malt beers were 49.9% and 16.1% higher than in two-row malt beers. Ethyl esters usually have a low threshold in beer and they can significantly affect the organoleptic properties of beer [40]. Ethyl laurate, palmitic acid ethyl ester, pentadecanoic acid ethyl ester, ethyl myristate, and ethyl 9-hexadecenoate exhibited the highest levels in MB, and it is clear that these ethyl esters provided MB with an aroma profile that was distinct from TB and MB.

Acids are mainly low molecular weight organic acids produced by yeast metabolism, which are important for the balance and crispness of beer [41]. Among the acids, decanoic acid and octanoic acid accounted for the total volatile acids (95.5–95.8%), and they can give the beer sweet and vinegary flavors, as well as being the main representative substances of the yeast flavor [42]. The contents of octanoic acid in the samples were 992.82 μg/L (SB), 912.14 μg/L (MB), and 917.10 μg/L (TB), respectively. The contents of decanoic acid in the samples were 171.16 μg/L(SB), 112.62 μg/L (MB), and 144.56 μg/L (TB), respectively. Both octanoic acid and decanoic acid had the highest content in SB, which may negatively affect the beer’s sensory quality.

In addition, acetaldehyde was the most abundant aldehyde in the beer and the highest detection in the sample [38]. Acetaldehyde is a carcinogen and its concentration should be controlled [43]; its contents in beer were 65.89 μg/L (SB), 62.83 μg/L (MB), and 68.19 μg/L(TB), respectively. The acetaldehyde content in MB and SB was lower than that of TB and it was lower in the beers, which suggests that beers produced by six-row malt can reduce the production of acetaldehyde to some extent.

#### 3.2.4. Effect of Different Row-Type Malt on the Ratio of Higher Alcohols to Esters

The ratio of higher alcohols to esters in beer is one of the critical indicators for evaluating beer quality. When the alcohol-to-ester ratio fell within the range of 3:1 to 5:1 [44], the beer exhibited a more harmonious and mellow flavor profile, achieving a better balance between a robust body and fruity aromatic characteristics [45,46]. Higher alcohols and esters were primarily derived from yeast metabolism during the fermentation process. The major higher alcohols included 1-propanol, 2-methyl-1-propanol, 3-methyl-1-butanol, and phenylethyl alcohol, while the primary esters consisted of ethyl acetate, ethyl butyrate, isoamyl acetate, and phenethyl acetate [45]. Analysis of beers brewed with different types of malt revealed significant differences in their alcohol-to-ester ratios. Specifically, the ratios for TB, MB, and SB were 4.92, 4.27, and 4.35, respectively. Among these, SB exhibited the lowest alcohol-to-ester ratio, resulting in a softer body and more pronounced fruity notes. In contrast, TB had the highest alcohol-to-ester ratio. These findings suggest that the use of six-row malt contributed to a softer beer flavor, while two-row malt tended to enhance the beer’s fullness and robustness.

### 3.3. Multivariate Analysis

#### 3.3.1. PLS-DA Analysis

**Figure 4:**
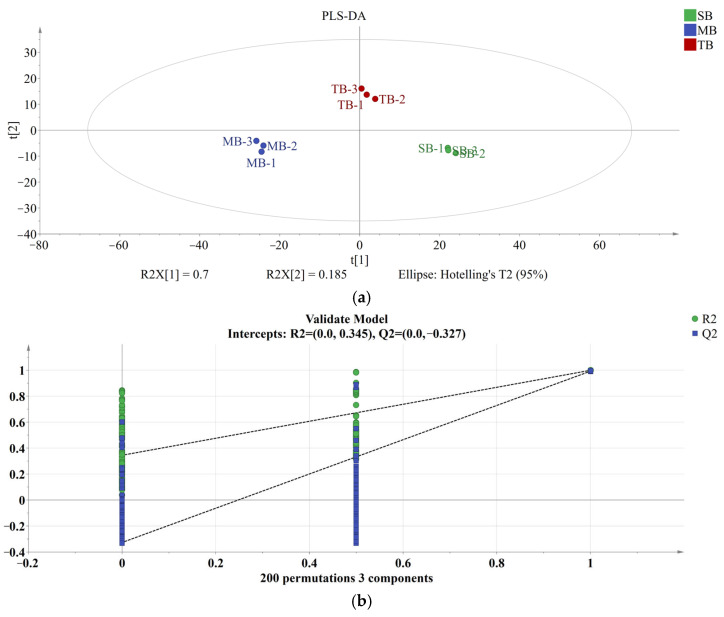

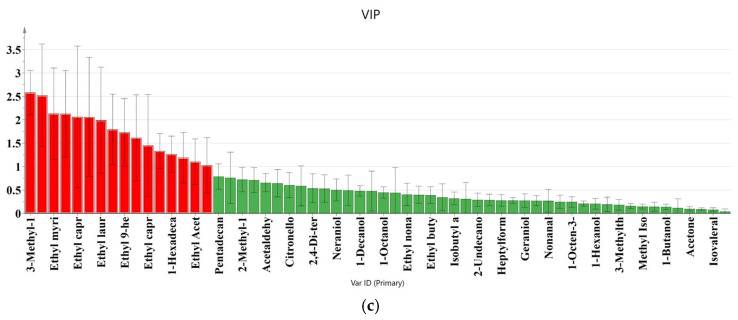
Partial least squares discriminant analysis (PLS-DA) of volatile compounds (VCs) in the three types of beer samples. Score scatter plot for the PLS-DA model (**a**); Permutation test (**b**) for the validation of the PLS-DA model; variables’ importance in projection (VIP) plots of PLS-DA model variables (**c**); the red color in the graph indicates that the VIP > 1.

To further reveal the effect of malt with different row-types on beer’s VCs, the differences between beer’s volatiles were analyzed by PLS-DA. PLS-DA, a multi-class discriminant classifier that uses sample variability to delineate boundaries between categories, has become one of the most commonly used discriminant techniques in the field of beer [47]. As shown in Figure 4a, the model had R2X = 0.973, R2Y = 0.991, and Q2 > 0.9, which indicated that the model was highly reliable. The intercept of the regression line on the *Y*-axis for the predicted values of Q^2^ after the 200 permutation tests was less than 0 (Figure 4b), indicating that the model was not overfitted. TB, MB, and SB were located in quadrants 1, 3, and 4, respectively, indicating that there were significant differences in the VCs of all three beers, which was in agreement with the results of the GC-E-nose, and the flavor heatmap analyses.

The variables’ importance in projection (VIP) score quantified the contribution of each compound to the model classification of the samples, and compounds with VIP > 1 played a key role in the classification of the samples [35]. Sixteen key differentially VCs were screened based on VIP > 1 (Figure 4c), including 3-methyl-1-butanol (VIP: 2.58), 2-ethylhexanol (VIP: 1.80), phenylethyl alcohol (VIP: 2.52), 1-hexadecanol (VIP: 1.27), ethyl acetate (VIP: 1.11), isoamyl acetate (VIP: 2.13), ethyl hexanoate (VIP: 1.19), ethyl caprylate (VIP: 1.45), ethyl caprate (VIP: 2.06), phenethyl acetate (VIP: 1.61), ethyl laurate (VIP: 1.99), palmitic acid ethyl ester (VIP: 1.03), ethyl 9-hexadecenoate (VIP: 1.73), ethyl myristate (VIP: 2.13), octanoic acid (VIP: 2.05), and decanoic acid (VIP: 1.33). Thus, the different row-types of malt affect the flavor of the beer mainly by influencing the content of these 16 compounds. However, further systematic analysis of the VCs was required to fully quantify and assess the contribution of each volatile compound to the overall beer’s VCs. 

#### 3.3.2. rOAV and ROAV Analysis

The true contribution of VCs to the overall aroma profile of a beer cannot be reflected by their relative amounts alone. To further understand the effect of different row-type malts on beer aroma, rOAV, and ROAV analyses were performed to evaluate the contribution of each volatile compound to the overall aroma. The greater the rOAV and ROAV of the VCs, the greater the contribution to the overall aroma profile of the sample [22]. In the samples, a total of fifteen VCs had rOAV > 1 and/or ROAV > 1, including four alcohols, eight esters, two aldehydes, and one acid (Table S5). It is noteworthy that the ROAV values of VCs in TB and SB were similar, and the ROAV values of VCs in MB were all lower, but the ROAVs of 2-ethylhexanol and phenylethyl acetate above 1 were only in MB.

**Figure 5:**
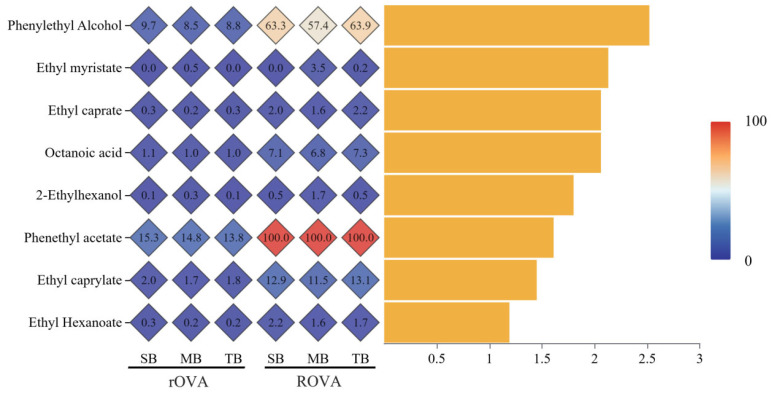
Point stick heatmap for the 8 key volatiles (VIP > 1, rOAV > 1, and/or ROAV > 1). The yellow column on the right represents the VIP value of the corresponding compound. The color mapping on the far right was to visually illustrate the difference in values of ROAV or rOAV.

A total of eight VCs had VIP > 1, rOAV > 1, and/or ROAV > 1 (Figure 5), which were apparently the key volatiles causing the flavor differences between the three beer samples. Among them, phenethyl acetate (rOAV_TB_ = 13.8, ROAV_TB_ = 100; rOAV_MB_ = 14.8, ROAV_MB_ = 100; rOAV_SB_ = 15.3, ROAV_SB_ = 100) was the volatile that contributed the most to the aroma of the three samples, which was mainly produced from phenylalanine via the Ehrlich pathway [48]. In addition, phenylethyl alcohol, ethyl caprylate, ethyl hexanoate, ethyl caprylate, and octanoic acid were also found to be the major flavor substances in the beers [49,50]. Phenylethyl alcohol, ethyl caprylate, and phenethyl acetate had the highest rOAV values in SB, which suggests that beers brewed with six-row malt may have strong floral and fruity aromas. Although ethyl hexanoate (fruity) and ethyl caprylate (fruity) had the highest rOAV values in TB, the higher content of octanoic acid may have masked their aroma, suggesting that the two-row malt beers may be more mellow. While in MB, except for 2-ethylhexanol and ethyl myristate, which had the highest ROAV values, the key flavor substances had the lowest ROAV values, suggesting that MB has more prominent nutty and oily aromas.

#### 3.3.3. Correlation Analysis

**Figure 6:**
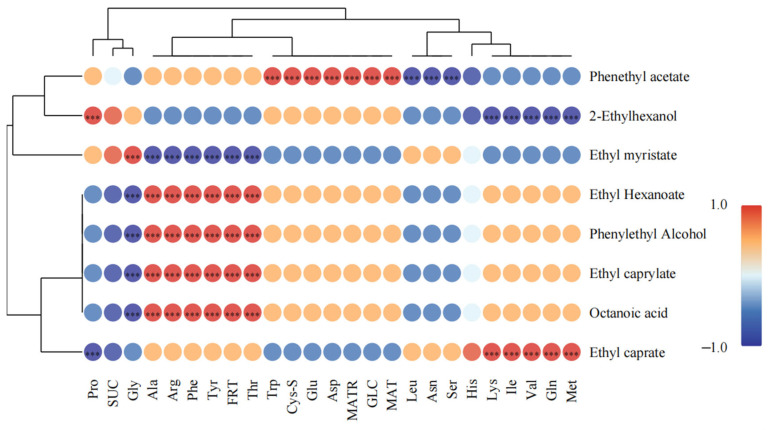
Heatmap of intergroup Spearman’s correlation. *** indicates that *p* < 0.05 and |r| > 0.7. Fructose (FRT); glucose (GLC); galtose (MAT); maltotriose (MTR); sucrose (SUC).

Intergroup correlation heatmaps were constructed based on Spearman’s correlation coefficient, and the intrinsic linkage of the key VCs and their precursors (FSs, FAAs) was preliminarily explored (Figure 6).

The key flavor substances in beer were influenced by multiple flavor precursors, which is consistent with previous studies [9]. Apparently, the malt with different rows caused differences in Gly, Ala, Arg, Phe, Tyr, Thr, and glucose, which in turn influenced key flavor substances (62.5%). Amino acids in wort are deaminated to keto acids, decarboxylated to aldehydes, reduced to the corresponding higher alcohols (Ehrlich pathway), and finally to the corresponding esters, and an increase in amino acid content promotes the formation of the corresponding alcohols, aldehydes, acids, and esters [9]. Taking phenylalanine as an example, it was previously reported that it could promote the formation of phenethyl acetate and phenylethyl alcohol [48], and the same phenomenon was observed in this study. Valine and leucine have been shown to generate the corresponding higher alcohols (2-methyl-1-propanol, 3-methyl-1-butanol, respectively) via the Ehrlich pathway [38]. Although 2-methyl-1-propanol and 3-methyl-1-butanol exhibited the highest content in SB, the content of valine and leucine in six-row malt wort was not the highest. It is noteworthy that SB exhibited the highest content of FSs, suggesting that elevated sugar levels may promote the formation of certain alcohols derived from FAAs, such as isobutanol and isoamyl alcohol. This phenomenon can be attributed to the fact that yeast synthesis of amino acids is accompanied by the production of α-keto acids, which can undergo a series of reactions to form corresponding VCs [51]. Nisbet et al. investigated the effect of glucose on VCs in wine by labeling glucose with C^13^ and found that about 90% of the VCs associated with amino acids could also be produced by glucose [52]. Although different flavor substances are affected by precursors differently, the effect of flavor precursors on VCs is mainly manifested as a promoting effect. Therefore, it is reasonable to speculate that a certain range of carbon and nitrogen sources in wort can drive the generation of flavor substances in beer.

## 4. Conclusions

In this study, the effect of different row-types of malt on beer VCs was investigated by GC-MS and GC-E-nose techniques. The results support the following: (i) there were significant differences in the composition and content of flavor precursors (FAAs, FSs) in wort from different row-types of malts; (ii) these differences manifested themselves as differences in VCs in the final beer; (iii) eight key volatile compounds were believed to be responsible for the differences in the beer’s distinctions; and (iv) the flavor precursors responsible for the differences in these eight substances were identified. This study clarified how different row-types of barley malt affect the VCs of beer and provides guidance for the selection of raw materials for beer production.

## Figures and Tables

**Table 1:** Relative content of flavor compounds in different beers (μg/L).

Class	Flavor Compounds	CAS	TB	MB	SB
Alcohols	1-Propanol	71-23-8	26.09 ± 4.80 a	21.29 ± 1.53 a	25.57 ± 0.94 a
	2-Methyl-1-propanol	78-83-1	107.39 ± 2.29 b	97.33 ± 3.87 c	115.15 ± 1.52 a
	1-Butanol	71-36-3	2.16 ± 0.05 b	1.78 ± 0.10 c	2.43 ± 0.11 a
	3-Methyl-1-butanol	123-51-3	1032.20 ± 22.15 b	936.55 ± 14.56 c	1146.50 ± 4.30 a
	1-Hexanol	111-27-3	3.49 ± 0.21 a	2.57 ± 0.01 b	3.63 ± 0.31 a
	1-Octen-3-ol	3391-86-4	1.25 ± 0.02 b	0.00 ± 0.00 c	1.52 ± 0.02 a
	2-Ethylhexanol	104-76-7	20.21 ± 0.48 c	77.63 ± 1.17 a	23.13 ± 0.04 b
	2-Nonanol	628-99-9	1.37 ± 0.13 b	1.01 ± 0.03 c	1.73 ± 0.25 a
	Linalool	78-70-6	37.25 ± 0.85 b	26.93 ± 0.64 c	42.79 ± 0.16 a
	1-Octanol	111-87-5	21.19 ± 0.58 b	18.76 ± 0.47 c	24.76 ± 0.02 a
	1-Nonanol	143-08-8	7.60 ± 0.22 b	6.60 ± 0.25 c	8.96 ± 0.17 a
	3(Z)-Nonenol	10340-23-5	3.07 ± 0.11 b	0.00 ± 0.00 c	3.25 ± 0.04 a
	3-Methylthiopropanol	505-10-2	2.62 ± 0.13 b	3.03 ± 0.27 a	2.91 ± 0.07 ab
	2-Undecanol	1653-30-1	4.26 ± 0.21 b	2.48 ± 0.05 c	4.70 ± 0.04 a
	1-Decanol	112-30-1	21.45 ± 0.56 b	18.34 ± 1.21 c	25.58 ± 0.50 a
	Citronellol	106-22-9	38.47 ± 1.30 b	30.96 ± 2.07 c	42.73 ± 0.88 a
	Neraniol	106-25-2	4.89 ± 0.20 a	0.00 ± 0.00 b	5.21 ± 0.28 a
	Geraniol	106-24-1	10.99 ± 0.34 b	9.37 ± 0.26 c	11.57 ± 0.20 a
	Phenylethyl Alcohol	60-12-8	1236.93 ± 27.65 b	1192.19 ± 17.44 b	1353.47 ± 22.07 a
	1-Hexadecanol	36653-82-4	0.00 ± 0.00 b	12.37 ± 1.04 a	11.84 ± 1.06 a
	T-cadinol	5937-11-1	10.24 ± 0.43 b	10.57 ± 0.48 b	12.06 ± 0.85 a
Total			2469.77 ± 11.27 c	2593.11 ± 7.52 b	2869.49 ± 28.52 a
Esters	Ethyl Acetate	141-78-6	75.37 ± 0.90 b	75.05 ± 1.43 b	94.07 ± 2.27 a
	Ethyl propionate	105-37-3	2.31 ± 0.10 c	2.75 ± 0.05 b	3.16 ± 0.17 a
	Isobutyl acetate	110-19-0	2.56 ± 0.10 c	3.05 ± 0.04 b	3.65 ± 0.17 a
	Ethyl butyrate	105-54-4	7.76 ± 0.13 b	7.62 ± 0.16 b	10.16 ± 0.29 a
	Isoamyl acetate	123-92-2	100.63 ± 4.23 c	120.14 ± 1.76 b	150.88 ± 6.17 a
	Ethyl Hexanoate	123-66-0	49.91 ± 0.89 b	49.84 ± 1.59 b	71.27 ± 1.58 a
	Ethyl heptanoate	106-30-9	1.91 ± 0.04 b	1.44 ± 0.11 c	2.28 ± 0.03 a
	2-Ethylhexyl acetate	103-09-3	0.93 ± 0.93 c	4.59 ± 0.23 a	2.42 ± 0.07 b
	Ethyl caprylate	106-32-1	524.22 ± 39.03 ab	494.24 ± 45.46 b	573.89 ± 9.39 a
	Heptylformiat	112-23-2	5.49 ± 0.23 a	3.94 ± 0.03 b	5.68 ± 0.06 a
	Ethyl nonanoate	123-29-5	11.52 ± 0.93 a	8.34 ± 1.11 b	11.33 ± 0.38 a
	Ethyl caprate	110-38-3	456.32 ± 31.98 a	366.73 ± 39.52 b	455.15 ± 5.99 a
	Octanoic acid isoamyl	2035-99-6	19.74 ± 1.79 a	16.63 ± 2.00 a	17.72 ± 0.43 a
	Phenethyl acetate	103-45-7	262.86 ± 12.28 b	281.81 ± 10.17 ab	290.31 ± 4.43 a
	Ethyl laurate	106-33-2	298.20 ± 10.11 b	354.15 ± 22.01 a	319.75 ± 1.63 b
	3-methylbutyl decanoate	2306-91-4	10.34 ± 0.61 ab	9.38 ± 0.89 b	10.84 ± 0.13 a
	Pentadecanoic acid, ethyl ester	41114-00-5	6.96 ± 0.19 c	15.75 ± 1.47 a	9.64 ± 0.80 b
	Palmitic acid ethyl ester	628-97-7	57.69 ± 0.93 b	76.74 ± 7.60 a	60.41 ± 3.69 b
	Ethyl 9-hexadecenoate	54546-22-4	29.34 ± 0.39 b	81.71 ± 6.16 a	33.65 ± 1.19 b
	Ethyl myristate	124-06-1	5.08 ± 0.04 b	93.05 ± 9.11 a	0.00 ± 0.00 b
Total			2066.96 ± 47.34 b	1929.16 ± 32.02 b	2126.27 ± 18.17 a
Acids	Acetic acid	64-19-7	14.83 ± 0.14 b	16.79 ± 1.44 b	22.41 ± 2.42 a
	6-Methylheptanoic acid	929-10-2	3.88 ± 0.29 b	5.18 ± 0.11 a	3.57 ± 0.10 b
	Octanoic acid	124-07-2	917.10 ± 37.44 b	912.15 ± 24.26 b	992.82 ± 6.79 a
	Decanoic acid	334-48-5	144.56 ± 1.22 b	112.62 ± 8.36 c	171.16 ± 2.53 a
	9-Decenoic acid	14436-32-9	29.61 ± 0.85 a	22.80 ± 3.01 b	29.24 ± 0.67 a
Total			1069.53 ± 32.54 b	1109.98 ± 38.16 b	1219.20 ± 5.72 a
Ketones	Acetone	67-64-1	6.84 ± 0.13 c	7.04 ± 1.01 b	8.59 ± 1.11 a
	Methyl Isobutyl Ketone	108-10-1	0.77 ± 0.03 b	0.63 ± 0.06 c	0.94 ± 0.05 a
	6-Methyl-5-hepten-2-one	110-93-0	1.49 ± 0.05 b	1.07 ± 0.05 c	1.75 ± 0.02 a
	2-Nonanone	821-55-6	2.84 ± 0.07 b	2.11 ± 0.03 c	3.53 ± 0.08 a
	Dihydropseudoionone	689-67-8	0.66 ± 0.05 b	0.51 ± 0.04 c	0.79 ± 0.05 a
Total			15.48 ± 0.52 b	17.81 ± 0.04 c	20.44 ± 0.34 a
Aldehydes	Acetaldehyde	75-07-0	12.04 ± 0.02 b	11.17 ± 0.55 c	13.43 ± 0.42 a
	Isobutyraldehyde	78-84-2	68.19 ± 0.59 a	62.83 ± 0.79 c	65.89 ± 0.53 b
	Isovaleraldehyde	590-86-3	0.33 ± 0.02 a	0.30 ± 0.01 a	0.32 ± 0.01 a
	Nonanal	124-19-6	0.56 ± 0.05 b	0.53 ± 0.02 b	0.68 ± 0.04 a
	2,5-Dimethylbenzaldehyde	5779-94-2	1.16 ± 0.29 b	1.63 ± 0.08 ab	1.95 ± 0.32 a
Total			70.72 ± 0.44 c	75.64 ± 0.50 b	78.13 ± 1.70 a
Others	Dimethyl sulfide	75-18-3	5.40 ± 0.19 a	5.43 ± 1.16 a	9.29 ± 1.04 a
	Caryophyllene oxide	1139-30-6	1.25 ± 0.09 b	1.33 ± 0.18 b	1.51 ± 0.27 a
	2,4-Di-tert-butylphenol	96-76-4	20.20 ± 0.51 b	11.59 ± 0.44 c	22.09 ± 0.34 a
	1,6-Dimethyl-4-isopropylnaphthaIene	483-78-3	5.77 ± 0.15 c	7.05 ± 0.70 b	9.08 ± 0.49 a
Total			27.01 ± 1.81 c	34.06 ± 0.45 b	41.27 ± 1.57 a

Note: a–c indicated significant differences between values at *p* < 0.05. TB: two-row malt beer; MB: mixed malt beer; SB: six-row malt beer.

## Data Availability

The data presented in this study are contained within the article.

## Supplementary Materials

The following supporting information can be downloaded at: https://www.mdpi.com/article/10.3390/foods14122010/s1, Table S1: the basic physicochemical parameters of wort; Table S2: the contents of fermentable sugars in wort (mg/mL); Table S3: the contents of free amino acids in wort (mg/mL); Table S4: the basic physicochemical parameters of beer sample; Table S5: the VIP, rOAV, and ROAV values of flavor compounds in beer.
