# Supplementary material for: Impact of Barley Malt with Different Row-Types on the Volatile Compounds in Beer
Source: Foods. 2025 Jun 6;14(12):2010. doi: 10.3390/foods14122010 (PMC12191998; doi:10.3390/foods14122010)
Supplement: Supplementary file 1 [file foods-14-02010-s001.zip › foods-3658753-supplementary.pdf]

Supplementary Tables

Table S1. The basic physicochemical parameters of wort.

Wort	TW	MW	SW
Plato (%)	12.00±0.00a	12.00±0.00a	12.00±0.00a
pH	5.58±0.01a	5.56±0.01ab	5.56±0.01b
α-N (mg/L)	229.38±3.15b	233.97±4.11b	244.63±4.22a
Reducing sugar (g/100 mL)	81.02±0.61b	83.54±1.76ab	85.01±1.42a
Color (EBC)	7.47±0.12a	6.87±0.21b	6.77±0.15b

Note: TW, MW, and SW represent two-rowed malt wort, mixed malt wort, and six-rowed malt wort, respectively; α-N: α-amino nitrogen; a-b: indicated significant differences between values at $p < 0.05$.

Table S2. The contents of fermentable sugars in wort (mg/mL)

Fermentable sugars		TW	MW	SW
	Fructose	1.16±0.01a	1.13±0.01b	1.17±0.01a
	Glucose	8.55±0.03c	9.34±0.07b	10.54±0.03a
	Maltose	3.09±0.05c	3.43±0.03b	3.94±0.07a
	Maltotriose	18.12±0.21c	20.63±0.01b	23.64±0.11a
	Sucrose	0.92±0.01a	0.93±0.00a	0.92±0.01a
Total		31.84±0.26c	35.46±0.06b	40.21±0.15a

Note: a-c: indicated significant differences between values at $p < 0.05$.

Table S3. The contents of free amino acids in wort (mg/mL)

Free amino acids		TW	MW	SW
I	Asp	52.2±1.0 b	52.3±0.6 b	55.8±0.4 a
	Glu	68.9±2.4 c	75.5±0.2 b	87.1±0.5 a
	Asn	45.1±0.6 a	41.1±0.3 b	41±0.3 b
	Ser	9.9±0.1 a	9.2±0.1 b	9.0±0.0 c
	Gln	15.5±0.3 a	14.3±0.2 c	15±0.1 b
	Thr	41.2±0.4 a	39.8±0.2 b	41.5±0.2 a
	Met	20.2±0.4 a	18.5±0.4 c	19.2±0.1 b
	Pro	172.4±15.1 a	196.6±7.1 a	174.8±29.3 a
II	Gly	21.8±0.4 b	25.7±0.8 a	21.3±0.3 b
	Tyr	58.7±0.3 b	57.4±0.0 c	59.7±0.8 a
	Val	70.8±1.1 a	67±0.7 b	68.1±0.3 b
	Phe	74.5±0.9 ab	73±0.8 b	75.5±0.5 a
	Ile	42.6±0.7 a	40.9±0.4 b	41.8±0.2 ab
III	His	29.2±2.4 a	27.9±1.6 a	27.9±3.3 a
	Arg	82.5±1.3 a	81.1±0.7 a	82.6±0.3 a
	Leu	108.0±2.0 a	99.9±1.0 b	99.6±0.3 b
	Lys	50.3±0.4 a	47.6±1.1 b	49.3±1.2 ab
Others	Ala	64.7±1.0 b	64.5±0.7 b	67.3±0.4 a
	Cys-S	1.2±0.0 c	1.3±0.0 b	1.8±0.0 a
	Trp	18.1±0.2 c	20.2±0.1 b	21.7±0.1 a
Total		1047.5±16.2a	1053.9±1.0a	1059.7±27.6a

Note: a-c: indicated significant differences between values at $p < 0.05$.

Table S4. The basic physicochemical parameters of beer sample.

Beer	TB	MB	SB
Ethanol (%vol)	4.22±0.02a	4.18±0.04a	4.22±0.05a
Real extract (%)	5.18±0.07a	5.01±0.01b	4.97±0.06b
RDF (%)	57.26±0.22c	57.83±0.30b	58.29±0.01a
pH	4.71±0.01a	4.61±0.01c	4.64±0.01b

Note: Different superscript letters in the row indicate significant differences between values at $p < 0.05$. TB: two-row malt beer; MB: mixed malt beer; SB: six-row malt beer; RDF: real fermentation degree.

Table S5. The VIP, rOAV, and ROAV values of flavor compounds in beer.

Class	Flavor compounds	Threshold[a]	VIP	rOAV			ROAV			Odor description[b]
		μg/L		TB	MB	SB	TB	MB	SB	
Alcohols	1-Propanol	800000	0.44	0.00	0.00	0.00	0.00	0.00	0.00	alcohol, candy, must, plastic, pungent
	2-Methyl-1-propanol	200000	0.72	0.00	0.00	0.00	0.00	0.00	0.00	alcohol, apple, cocoa, fusel, glue
	1-Butanol	459.2	0.14	0.01	0.00	0.00	0.03	0.03	0.03	alcohol, caramel, fermented, fragant, fruit
	3-Methyl-1-butanol	70000	2.58	0.02	0.01	0.01	0.11	0.09	0.11	alcohol, balsamic, banana, cheese, cocoa
	1-Hexanol	4000	0.21	0.00	0.00	0.00	0.01	0.00	0.01	bread, fat, flower, fruit, grass
	1-Octen-3-ol	100	0.25	0.02	0.00	0.01	0.10	0.00	0.09	earth, fat, fish, floral, grass
	2-Ethylhexanol	300	1.80	0.08	0.26	0.07	0.50	1.74	0.49	citrus, green, oil, rose
	2-Nonanol	420	0.14	0.00	0.00	0.00	0.03	0.02	0.02	coconut, cucumber, fruit, pungent, wax
	Linalool	6	0.71	7.13	4.49	6.21	46.68	30.26	44.88	bergamot,

Class	Flavor compounds	Threshold[a] µg/L	VIP	rOAV TB	MB	SB	ROAV TB	MB	SB	Odor description[b]
										coriander, floral, flower, grape
	1-Octanol	125.8	0.45	0.20	0.15	0.17	1.29	1.01	1.22	citrus, detergent, fat, fruit, green
	1-Nonanol	1000	0.28	0.01	0.01	0.01	0.06	0.04	0.05	detergent, dust, fat, floral, green
	3(Z)-Nonenol	-	0.40							floral, green, pungent
	3-Methylthiopropanol	250	0.18	0.01	0.01	0.01	0.08	0.08	0.08	burnt milk, caramel, cooked potato, earth, garlic
	2-Undecanol	41	0.29	0.11	0.06	0.10	0.75	0.41	0.75	
	1-Decanol	2800	0.48	0.01	0.01	0.01	0.06	0.04	0.06	fat, oil, orange, plastic
	Citronellol	1000	0.60	0.04	0.03	0.04	0.28	0.21	0.28	citronella, citrus, floral, green, lemon
	Neraniol	2200	0.50	0.00	0.00	0.00	0.02	0.00	0.02	citrus, floral, fruit, lemon, rose
	Geraniol	100	0.27	0.12	0.09	0.11	0.76	0.63	0.79	citrus, floral, geranium, lemon peel, metal

Class	Flavor compounds	Threshold[a]	VIP	rOAV			ROAV			Odor description[b]
		μg/L		TB	MB	SB	TB	MB	SB	
	Phenylethyl Alcohol	140	2.52	9.67	8.52	8.84	63.27	57.41	63.86	corn flakes, floral, fruit, honey, rose
	1-Hexadecanol	-	1.27							flower, wax
	T-cadinol	-	0.34							astringent, sweet, wood
Esters	Ethyl Acetate	21000	1.11	0.00	0.00	0.00	0.03	0.02	0.03	balsamic, butter, contact glue, fruit, grape
	Ethyl propionate	160	0.29	0.02	0.02	0.01	0.13	0.12	0.10	apple, balsamic, fruit, pineapple, rum
	Isobutyl acetate	1600	0.32	0.00	0.00	0.00	0.01	0.01	0.01	apple, banana, floral, fruit, herb
	Ethyl butyrate	367	0.39	0.03	0.02	0.02	0.18	0.14	0.15	anise, apple, banana, butter, fruit
	Isoamyl acetate	1200	2.13	0.13	0.10	0.08	0.82	0.67	0.61	apple, banana, fresh, fruit, glue
	Ethyl Hexanoate	210	1.19	0.34	0.24	0.24	2.22	1.60	1.72	anise, apple peel, banana, brandy, cheese
	Ethyl heptanoate	1.9	0.16	1.20	0.76	1.00	7.85	5.12	7.26	banana, brandy, fruit, strawberry,

Class	Flavor compounds	Threshold[a] μg/L	VIP	rOAV TB	rOAV MB	rOAV SB	ROAV TB	ROAV MB	ROAV SB	Odor description[b]
										wine
	2-Ethylhexyl acetate	260	0.53	0.01	0.02	0.00	0.06	0.12	0.03	
	Ethyl caprylate	290	1.45	1.98	1.70	1.81	12.95	11.49	13.07	apple, apricot, banana, brandy, fresh
	Heptylformiat	-	0.28							
	Ethyl nonanoate	19.3	0.40	0.59	0.43	0.60	3.84	2.91	4.32	banana, fruit, grape
	Ethyl caprate	1500	2.06	0.30	0.24	0.30	1.99	1.65	2.20	brandy, burnt, fruit, geranium, grape
	Octanoic acid isoamyl	70	0.48	0.25	0.24	0.28	1.66	1.60	2.04	baked apple
	Phenethyl acetate	19	1.61	15.28	14.83	13.83	100.00	100.00	100.00	floral, fruit, honey, rose, tobacco
	Ethyl laurate	5900	1.99	0.05	0.06	0.05	0.35	0.40	0.37	cream, floral, fruit, green apple, leaf
	3-methylbutyl decanoate	-	0.19							fat, wax
	Pentadecanoic acid, ethyl ester	-	0.79							
	Palmitic acid ethyl ester	1000	1.03	0.06	0.08	0.06	0.40	0.52	0.42	fat, fruit, rancid, sweet, wax

Class	Flavor compounds	Threshold[a]	VIP	rOAV			ROAV			Odor description[b]
		μg/L		TB	MB	SB	TB	MB	SB	
	Ethyl 9-hexadecenoate	-	1.73							
	Ethyl myristate	180	2.13	0.00	0.52	0.03	0.00	3.49	0.20	cream, ether, nut, oil, pleasant
Acids	Acetic acid	200000	0.76	0.00	0.00	0.00	0.00	0.00	0.00	acid, cheese, fruit, pungent, sour
	6-Methylheptanoic acid	-	0.25							
	Octanoic acid	910	2.06	1.09	1.00	1.01	7.14	6.76	7.28	acid, carrot, cheese, fat, fermented
	Decanoic acid	10000	1.33	0.02	0.01	0.01	0.11	0.08	0.10	dust, fat, grass, rancid, soap
	9-Decenoic acid	-	0.59							
Ketones	Acetone	832	0.10	0.00	0.00	0.00	0.01	0.01	0.01	chemical, ether, fruit, glue, hay
	Methyl Isobutyl Ketone	80	0.15	0.02	0.01	0.02	0.14	0.09	0.14	fruit, strawberry, sweet, varnish
	6-Methyl-5-hepten-2-one	68	0.21	0.05	0.03	0.04	0.34	0.21	0.30	citrus, fruit, mushroom, pepper, rubber
	2-Nonanone	82	0.09	0.01	0.01	0.01	0.06	0.04	0.06	fragant, fruit, green, hot milk, oil
	Dihydropseudoionone	-	0.27							

Class	Flavor compounds	Threshold[a]	VIP	rOAV			ROAV			Odor description[b]
		μg/L		TB	MB	SB	TB	MB	SB	
Aldehydes	Acetaldehyde	25000	0.66	0.00	0.00	0.00	0.02	0.02	0.02	ether, floral, fruit, green apple, pungent
	Isobutyraldehyde	1.5	0.04	0.21	0.20	0.22	1.40	1.35	1.60	burnt, caramel, cocoa, floral, fresh
	Isovaleraldehyde	0.15	0.08	4.55	3.56	3.75	29.78	24.00	27.13	acrid, almond, chocolate, cocoa, corn flakes
	Nonanal	260	0.27	0.01	0.01	0.00	0.05	0.04	0.03	citrus, cucumber, fat, floral, green
	2,5-Dimethylbenzaldehyde	200	0.49	0.05	0.03	0.03	0.30	0.18	0.20	
Others	Dimethyl sulfide	-	0.12							aonori, boiled onion, cabbage, gasoline, organic
	Caryophyllene oxide	410	0.64	0.05	0.03	0.05	0.35	0.19	0.36	citrus, fruit, herb, must, spice
	2,4-Di-tert-butylphenol	500	0.54	0.02	0.01	0.01	0.12	0.10	0.08	
	1,6-Dimethyl-4-isopropylnaphthaIene	-	0.31							

Note: The flavor and threshold of all flavor compounds were obtained from relevant literature and websites. VIP: variables' importance in projection -: means not detected. a: Odor detection threshold value in water or alcohol (μg/L) was obtained from the literature and a database: www.vcf-online.nl. c: Odor descriptions were taken from www.vcf-online.nl.
